# Genomics-Guided Analysis of NAD Recycling Yields Functional Elucidation of COG1058 as a New Family of Pyrophosphatases

**DOI:** 10.1371/journal.pone.0065595

**Published:** 2013-06-12

**Authors:** Lucia Cialabrini, Silverio Ruggieri, Marat D. Kazanov, Leonardo Sorci, Francesca Mazzola, Giuseppe Orsomando, Andrei L. Osterman, Nadia Raffaelli

**Affiliations:** 1 Department of Agricultural, Food and Environmental Sciences, Polytechnic University of Marche, Ancona, Italy; 2 A. A. Kharkevich Institute for Information Transmission Problems, Russian Academy of Sciences, Moscow, Russia; 3 Department of Clinical Sciences, Polytechnic University of Marche, Ancona, Italy; 4 Sanford-Burnham Medical Research Institute, La Jolla, California, United States of America; Instituto de Biociencias - Universidade de São Paulo, Brazil

## Abstract

We have recently identified the enzyme NMN deamidase (PncC), which plays a key role in the regeneration of NAD in bacteria by recycling back to the coenzyme the pyridine by-products of its non redox consumption. In several bacterial species, PncC is fused to a COG1058 domain of unknown function, highly conserved and widely distributed in all living organisms. Here, we demonstrate that the PncC-fused domain is endowed with a novel Co^+2^- and K^+^-dependent ADP-ribose pyrophosphatase activity, and discuss the functional connection of such an activity with NAD recycling. An in-depth phylogenetic analysis of the COG1058 domain evidenced that in most bacterial species it is fused to PncC, while in α- and some δ-proteobacteria, as well as in archaea and fungi, it occurs as a stand-alone protein. Notably, in mammals and plants it is fused to FAD synthase. We extended the enzymatic characterization to a representative bacterial single-domain protein, which resulted to be a more versatile ADP-ribose pyrophosphatase, active also towards diadenosine 5′-diphosphate and FAD. Multiple sequence alignment analysis, and superposition of the available three-dimensional structure of an archaeal COG1058 member with the structure of the enzyme MoeA of the molybdenum cofactor biosynthesis, allowed identification of residues likely involved in catalysis. Their role has been confirmed by site-directed mutagenesis.

## Introduction

**Figure 1:**
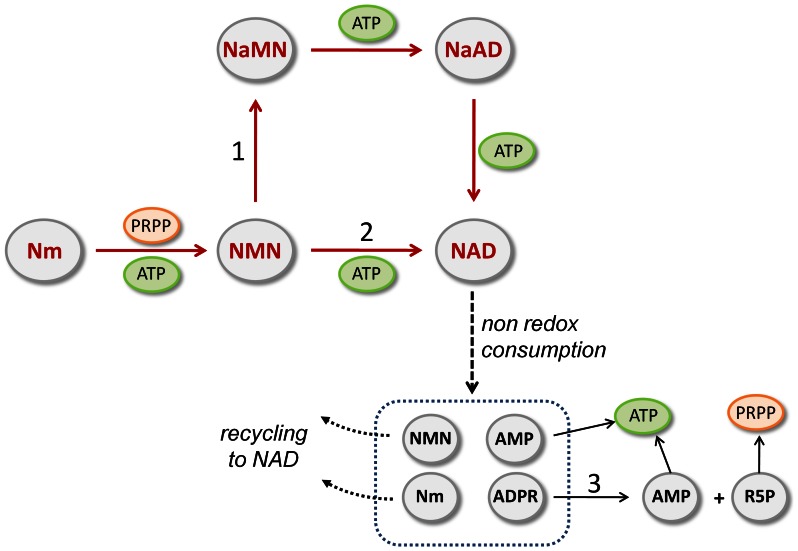
Recycling of bacterial NAD catabolism products. Reactions described in this study, numbered from 1 to 3, are catalyzed by: 1) NMN deamidase (PncC); 2) NMN adenylyltransferase of the NadM family; 3) ADPR pyrophosphatase. In several bacterial species PncC and NadM occur in fused forms with COG1058 ADPRP and Nudix ADPRP, respectively, as discussed in this work. Abbreviations: Nm, nicotinamide; NMN, nicotinamide mononucleotide; NaMN, nicotinate mononucleotide; NaAD, nicotinate adenine dinucleotide.

NAD is an ubiquitous and essential coenzyme involved in a huge number of redox reactions in all forms of cellular life. In addition, NAD is utilized as a co-substrate in a variety of non redox reactions playing an important role in DNA replication, DNA repair, RNA ligation, cell differentiation, and cellular signal transduction [1], [2], [3]. Specific enzymes catalyze the transfer of the NAD ribonucleotidyl moiety, either in the form of AMP or ADP-ribose (ADPR), to different functional groups of proteins and nucleic acids, thereby modulating their function. NAD-dependent ADP-ribosylation and deacetylation of various target proteins, as well as NAD-dependent dephosphorylation of tRNA are well known in eukaryotes. In contrast, non redox NAD-dependent processes in bacteria are still relatively unexplored. Only few bacterial NAD-consuming enzymes have been so far characterized: i) the NAD-dependent DNA ligase, which uses the AMP moiety of NAD to activate the 5′-phosphate of nicked DNA ends; ii) the NAD-dependent deacetylase CobB of the Sirt2 family, which catalyzes protein deacetylation by transferring ADPR from NAD to the acetyl group, with the release of O-acetyl-ADPR [4]; iii) various mono ADP-ribosyltransferases, which catalyze the covalent attachment of single ADPR units to both endogenous and host proteins to regulate their function [5], [6]; iv) an ortholog of the yeast tRNA 2′-phosphotransferase, able to catalyze tRNA dephosphorylation by transferring ADPR from NAD to the 2′-phosphate group of tRNA, with the release of 1,2 cyclic phosphate ADPR [7]. The occurrence of an intensive NAD consumption in bacteria is suggested by the remarkably rapid turnover of the intracellular NAD pool, which also emphasizes the importance of the continuous replenishing of the dinucleotide [8]. Notably, all the products of NAD consumption can contribute to its regeneration (Figure 1). In particular, the pyridine by-products, nicotinamide mononucleotide (NMN) and nicotinamide (Nm), can be recycled back to NAD through various recycling pathways that, in recent years, have begun to be elucidated in the majority of bacterial species, thanks to genomics-guided approaches [9], [10], [11], [12]. In turn, the ADPR moiety released from ADP-ribosylated proteins, or deriving from O-acetyl-ADPR and 1,2 cyclic phosphate ADPR, can be further hydrolyzed by ADPR pyrophosphatases (ADPRP) of the Nudix family, yielding AMP and ribose-5-phosphate, which may be reused in NAD biogenesis via conversion to ATP and PRPP, respectively, as already proposed in eukaryotes [13], [14], [15]. In this view, the occurrence in some bacterial species of a Nudix ADPRP domain fused to the enzyme NMN adenylyltransferase (NadM) provides evidence of the strict link between NAD consumption and regeneration (Figure 1) [16], [17].

**Figure 2:**
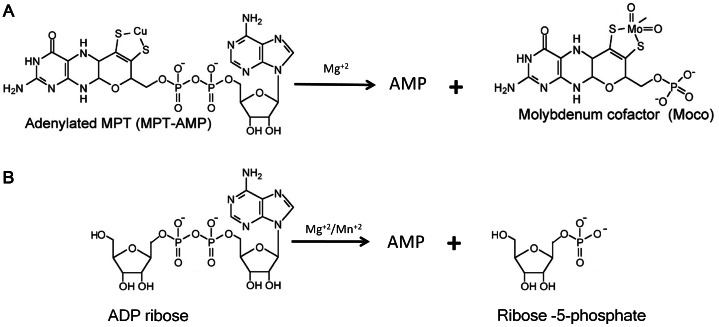
Pyrophosphatase reactions catalyzed by bacterial MoeA and its eukaryotic ortholog (A) and ADP-ribose pyrophosphatase (B). Both substrates share an adenosine group linked to two different moieties through the pyrophosphate bridge which is cleaved during the enzyme-catalyzed reaction.

Very recently, combining comparative genomic analysis, metabolic pathways reconstruction, and experimental characterization, we have identified the enzyme NMN deamidase (PncC), which converts NMN to nicotinate mononucleotide (NaMN), thus channeling the mononucleotide towards the deamidated NAD biosynthetic pathway (Figure 1) [10]. This enzyme plays a key role in NMN and Nm recycling back to NAD in the majority of bacterial species. Very often, PncC is found fused to a domain of unknown function, which belongs to the family of proteins classified as COG1058 in the Clusters of Orthologous Groups database, currently annotated as “predicted nucleotide-utilizing enzyme family, related to molybdopterin-biosynthesis enzyme MoeA” (PF00994). Members of this family share a high similarity with bacterial MoeA and its eukaryotic orthologues (*i.e.* the E-domains of mammalian gephyrin and plant Cnx1), which are involved in the last step of Molybdenum Cofactor (MoCo) biosynthesis [18]. In particular, these enzymes bind both molybdate and the adenylated form of cyclic pyranopterin monophosphate (MPT-AMP), and catalyze MPT-AMP hydrolysis, releasing AMP, with the concomitant insertion of molybdenum into MPT, yielding the active product MoCo [19]. Based on the catalyzed reaction, they have been considered to act like the enzyme ADPRP of the Nudix hydrolase family [19] (Figure 2). This suggestion, together with the observed tendency of ADPRP to associate with enzymes of NAD biosynthesis, led us to hypothesize that members of COG1058 might be endowed with ADPRP activity.

In this work, we demonstrated the ADPRP activity of the bacterial COG1058 domain, and provided evidence that COG1058 represents a novel pyrophosphatase family.

## Materials and Methods

### Cloning, Expression, and Protein Purification

The *COG1058* gene of *A. tumefaciens* was amplified by polymerase chain reaction (PCR) from genomic DNA and cloned into the pET100/D-TOPO vector (INVITROGEN Champion™ pET Directional TOPO® Expression Kits) according to manual’s instructions. Sequences of the synthetic oligonucleotides used as primers are reported in Table S1. The construct was sequence-verified for accuracy and used to transform *E.coli* BL21(DE3) cells for protein expression. Cells were grown at 37°C in Luria Bertani medium supplemented with 0.1 mg/ml ampicillin. After reaching an A_600_ of 0.6, expression was induced with 1 mM isopropyl β-D-thiogalactopyranoside. After 3 h induction at 37°C, the cells were harvested by centrifugation at 5,000×*g* for 10 min, at 4°C. All subsequent steps were performed at 4°C. Induced cells were resuspended in one-twentieth of the original culture volume with buffer A (50 mM TRIS/HCl buffer, pH 8.0, 1 mM MgCl_2_, 0.2 mM EDTA, 10 mM Imidazole) containing 1 mM phenylmethylsulfonyl fluoride and 0.002 mg/ml leupeptin, antipain and chymostatin. The suspension was sonicated for 3 min at 50 watt, with 30 sec intervals, and centrifuged at 15,000×*g* for 30 min. The supernatant deriving from 40 ml culture was applied to a 1-ml HisTrap HP column (GE Healthcare), equilibrated with buffer A. The column was washed with 30 mM Imidazole in buffer A, and elution was performed with an Imidazole gradient from 30 mM to 500 mM in buffer A. Fractions containing the recombinant protein (eluted at about 100 mM Imidazole) were pooled and purity of the preparation was assessed by sodium dodecyl sulfate polyacrylamide gel electrophoresis [20]. The pool (0.2 mg/ml protein concentration, as determined by using bovine serum albumin as the standard [21]), resulted to be stable for several months at 4°C, and was used for the catalytic characterization.

*S. oneidensis* COG1058/PncC protein was obtained as described in [10].

### Site-directed Mutagenesis

Site-directed mutagenesis of *A. tumefaciens* COG1058 was carried out using the QuikChange Site-Directed Mutagenesis Kit (Agilent Technologies). Sequences of mutagenic primers are reported in Table S1. pET100/D-TOPO recombinant plasmid was used as the template for the PCR mutagenesis reactions, by following kit’s instructions. The mutants were sequenced to verify incorporation of the desired modification and to ensure the absence of random mutations. For mutants expression, the mutagenized plasmids were transformed into *E.coli* BL21(DE3) cells and expression and purification of the mutated proteins were performed as for the wild-type protein.

### Pyrophosphatase Activity Assays

Activity was assayed by measuring the formation of the mononucleotides deriving from the pyrophosphate bond hydrolysis of the tested compounds. Nucleotides were separated by HPLC on a system equipped with a diode-array detector. The reaction mixtures contained 0.8 µg/ml *S. oneidensis* or 0.08 µg/ml *A.tumefaciens* purified recombinant protein, in 100 mM TRIS/HCl buffer, pH 7.5, 100 mM KCl, 0.1 mg/ml bovine serum albumin, 1 mM CoCl_2_, 0.5 mM substrate. After incubation at 37°C, reactions were stopped and subjected to HPLC analysis, using different procedures depending on the tested substrate. In particular, when FAD was tested as the substrate, reactions were stopped by adding formic acid (1∶20 of final assay volume). When NADH and NADPH were tested, reactions were stopped with 0.12 M NaOH; after 10 min on ice the samples were centrifuged for 6 min at 12,000×*g* and the supernatants were neutralized with 0.01 M HCl. In both cases, the samples were loaded onto a Phenomenex C18 Kinetex column (2.6 µm, 4.6×150 mm). For FMN determination, elution conditions were as follows: 5 min at 100% buffer A (100 mM potassium phosphate, pH 3.0, 10% methanol), 15 min up to 100% buffer B (100 mM potassium phosphate, pH 3.0, 30% methanol), holding at 100% buffer B for 5 min, returning to 100% buffer A in 1 min, and holding at 100% buffer A for 12 min. Flow rate was maintained at 0.5 ml/min, and temperature was fixed at 25°C. For NMNH determination, column was eluted as described above using buffer A consisting of 100 mM potassium phosphate, pH 6.0 and buffer B consisting of buffer A, containing 20% methanol. For the determination of the mononucleotides produced from all other tested substrates, reactions were stopped with 0.6 M HClO_4_; after 10 minutes on ice the samples were centrifuged for 6 min at 12,000×*g* and the supernatants were neutralized with 0.8 M K_2_CO_3_, kept on ice for 10 min and centrifuged as described above. Nucleotide separation was performed on a Supelco LC-18-T column (5 µm, 4.6×250 mm) at 18°C. Elution conditions were as described in [22]. In all assays, the amount of enzyme used ensured a substrate consumption below 5% of the initial concentration after a 10 min incubation. In addition, withdrawals from the assay mixtures at two different incubation times were always performed to ensure a linear time frame. Controls without enzymes were always processed in parallel to correct for the non-enzymatic, metal-ion catalyzed hydrolysis of several substrates. All measurements were performed in duplicate. Kinetic values were determined by fitting initial velocity data to the standard Michaelis-Menten equation using GraphPad Prism software package.

### Bioinformatics Tools and Resources

The COG1058 protein sequences in available complete genomes were taken from The SEED comparative genomics database [23]. Due to the large number of sequences retrieved, a special procedure had to be used for the construction of multiple sequence alignment: i) an approximate phylogenetic tree was built by the FastTree tool [24]; ii) all sequences were divided into fifteen clusters corresponding to the separate branches of the tree; iii) multiple alignment of sequences belonging to the same cluster was obtained using Clustal Omega [25]; iv) poorly aligned regions were cut from the cluster alignments; v) the final alignment was constructed using the profile-to-profile alignment option of the Clustal Omega algorithm. The phylogenetic tree was built by RAxML [26]. The species tree was taken from the Superfamily database [27]. Visualization of protein three-dimensional structures and structure comparison were performed using Chimera [28]. Multiple sequence alignment figures were prepared using TeXshade [29]. Genome context analysis was performed in The SEED environment.

## Results

### Bacterial Members of the COG1058 Family are Endowed with ADP-ribose Pyrophosphatase Activity

**Figure 3:**
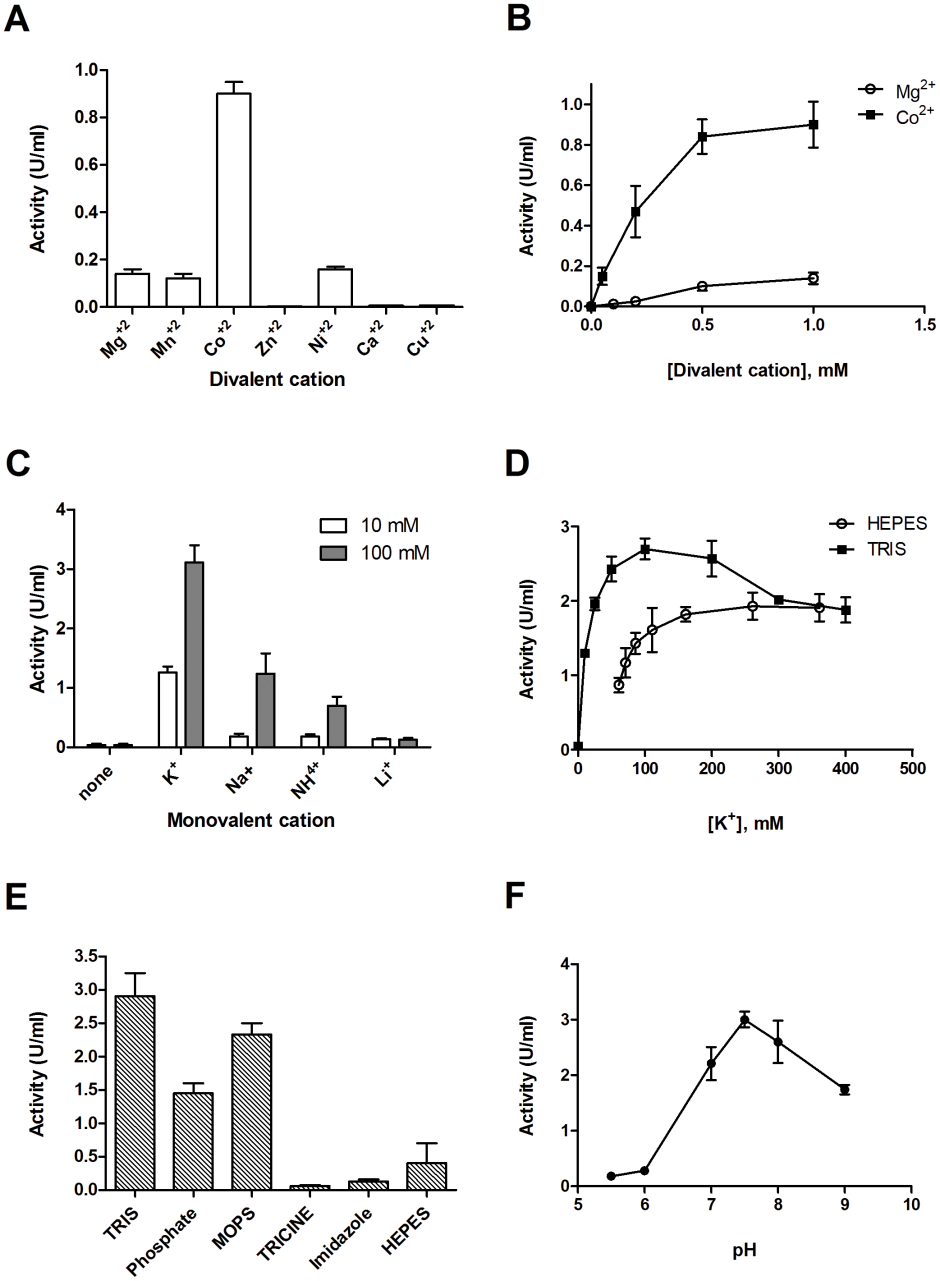
Characterization of ADP-ribose hydrolysis by recombinant *A. tumefaciens* COG1058 enzyme. Enzymatic assays were performed in the presence of 0.5 mM ADPR and 10 ng of pure protein. Reaction mixtures were incubated for 10 min at 37°C in: A) 100 mM HEPES/KOH, pH 7.5, in the presence of different divalent cations at 1 mM concentration (all ions were added as chloride salts); B) 100 mM HEPES/KOH, pH 7.5, with different concentrations of MgCl_2_ or CoCl_2_; C) 100 mM TRIS/HCl buffer, pH 7.5, 1 mM Co^+2^, in the presence of 10 mM and 100 mM of the indicated monovalent cations (added as chloride salts); D) 100 mM TRIS/HCl, pH 7.5 and 100 mM HEPES/KOH, pH 7.5, 1 mM Co^+2^, in the presence of different K^+^ concentrations (K^+^ ions were added as KCl); E) different buffer species at 100 mM concentration, pH 7.5, 1 mM Co^+2^, 0.1 M K^+^; F) 100 mM BIS-TRIS buffer at varying pH values, 1 mM Co^+2^, 0.1 M K^+^. One Unit of enzyme activity represents the amount of enzyme catalyzing the formation of 1 µmol of product per min, under the specified conditions.

Both *Shewanella oneidensis (So)* COG1058/PncC protein, in which the COG1058 domain is fused with the NMN deamidase (PncC) domain, and *A. tumefaciens* (*At*) COG1058 protein (gi 159184889), which comprises only the COG1058 domain, were assayed for the ADPRP activity. Both proteins were found to possess such activity in HEPES/KOH buffer, pH 7.5, 1.0 mM Mg^+2^. The ADPRP activity of the *At* enzyme was further characterized in order to determine the optimal conditions for the reaction. Catalysis resulted to be metal-dependent (Figure 3A). Among the tested divalent cations, Co^+2^ was the most effective in supporting the enzyme activity, with Ni^+2^, Mg^+2^ and Mn^+2^ being about seven-fold less efficient; 1 mM Ca^+2^, Cu^+2^ and Zn^+2^ did not sustain the activity at all (Figure 3A). Mg^+2^ and Co^+2^ titration experiments showed that the enzyme-catalyzed ADPR hydrolysis was optimal at 0.5 mM Co^+2^ (Figure 3B), while 10 mM Mg^+2^ was needed to reach the maximum activity, corresponding to about 30% of the optimal Co^+2^-dependent activity (not shown). We found that the presence in the reaction mixtures of a monovalent cation was also essential for the catalytic activity; replacement of HEPES/KOH buffer with TRIS/HCl abolished the enzymatic activity unless a monovalent cation was present (Figure 3C). Among the cations tested, K^+^ was the most effective (Figure 3C). The K^+^-dependence of the reaction velocity in the presence of either TRIS/HCl or HEPES/KOH buffers at pH 7.5, is shown in Figure 3D. Maximum activation was reached at different K^+^ concentrations depending on the buffering species: in TRIS/HCl buffer, 100 mM K^+^ was the most effective, whereas in HEPES/KOH buffer, maximum activity was reached at about 200 mM K^+^ concentration. As shown in Figures 3D and 3E, ADPR hydrolysis is significantly affected also by the buffering species present in the reaction mixture. Among the buffers tested at pH 7.5, in the presence of 100 mM K^+^, TRIS buffer was the best at sustaining activity, followed by MOPS, HEPES, and Phosphate. In the presence of Imidazole and TRICINE, a very low activity was measured (Figure 3E). Optimal pH was determined by measuring ADPR hydrolysis in a 50 mM BIS-TRIS/TRIS buffer system at pH values ranging from 5.5 to 8.5. Activity was optimal in a narrow range around pH 7.5 (Figure 3F).

The same Co^+2^-dependence and K^+^-activation, as well as pH optimum and buffering species dependence, were displayed by the *So*COG1058/PncC enzyme (not shown).

### Substrate Specificity Screening Reveals that ADP-ribose is the Preferred Substrate of Bacterial COG1058 Enzymes

**Figure 4:**
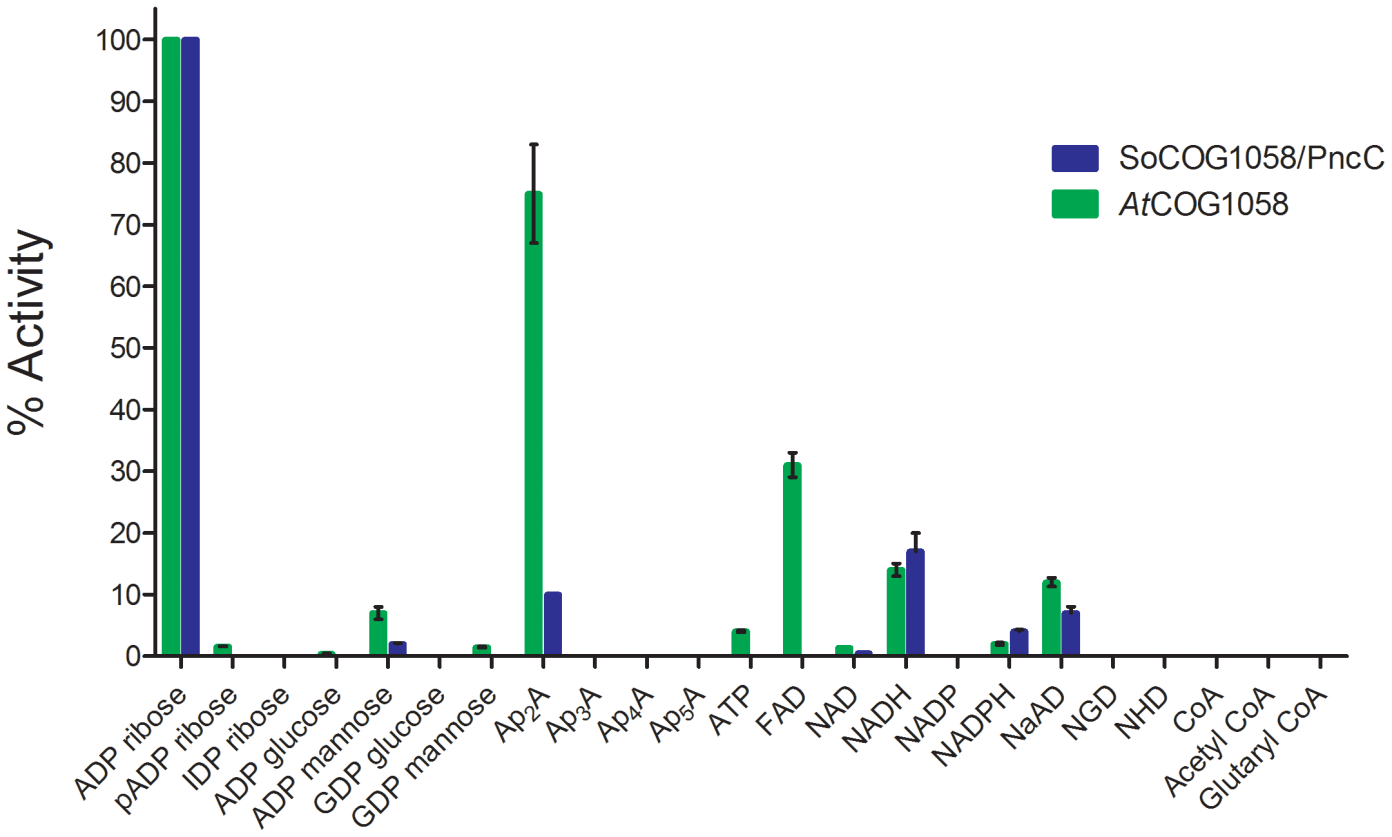
Substrate specificity screening of *At*COG1058 and *So*COG1058/PncC pyrophosphatases. The pyrophosphatase activity of the pure recombinant enzymes was assayed as described in “Materials and Methods”, in the presence of the listed compounds at 0.5 mM concentration each. Abbreviations: Ap_3_A, diadenosine triphosphate; Ap_4_A, diadenosine tetraphosphate; Ap_5_A, diadenosine pentaphosphate; NGD, nicotinamide guanine dinucleotide; NHD, nicotinamide hypoxanthine dinucleotide.

To get a deeper insight into the COG1058 domain substrate specificity, we performed a detailed *in vitro* screening of several compounds containing a pyrophosphate bond as potential substrates of *At*COG1058 and *So*COG1058/PncC enzymes, by using the assay conditions previously optimized towards ADPR. The results of the screening performed with the two enzymes are shown in Figure 4. Both enzymes display a Co^+2^-dependent pyrophosphatase activity towards a limited set of substrates, with ADPR being the preferred among the tested compounds. The stand-alone domain also hydrolyzes diadenosine 5′-diphosphate (Ap_2_A) to a significant extent (75% rate with respect to ADPR substrate), and shows some activity with FAD (30% rate), NADH and nicotinate adenine dinucleotide (NaAD) (14% rate). A very low, but still detectable activity is displayed by this enzyme towards NADPH and NAD, while NADP is not a substrate. The *So*COG1058/PncC bifunctional enzyme is more strictly specific for ADPR; in fact it hydrolyzes Ap_2_A at about 10% rate with respect to ADPR, while FAD is not a substrate. On the other hand, it behaves similarly to the *At*COG1058 enzyme towards the pyridine dinucleotides. For both enzymes, replacement of the ADP-linked ribose with mannose significantly decreases the activity, which is further decreased or completely abolished when ribose is replaced by glucose (Figure 4). A very low activity is displayed by the *At*COG1058 enzyme towards GDP-mannose, while GDP-glucose is not recognized by either protein. The presence of a phosphate group on the adenine-linked ribose significantly decreases or fully abolishes the activity. As to the Ap_n_A series, for both enzymes the activity falls off when n >2. ATP is hydrolyzed to AMP only by the *At*COG1058 protein and to a very low extent (Figure 4). Neither enzyme is able to hydrolyze the pyrophosphate bond in ribonucleoside diphosphates (not shown).

**Figure 5:**
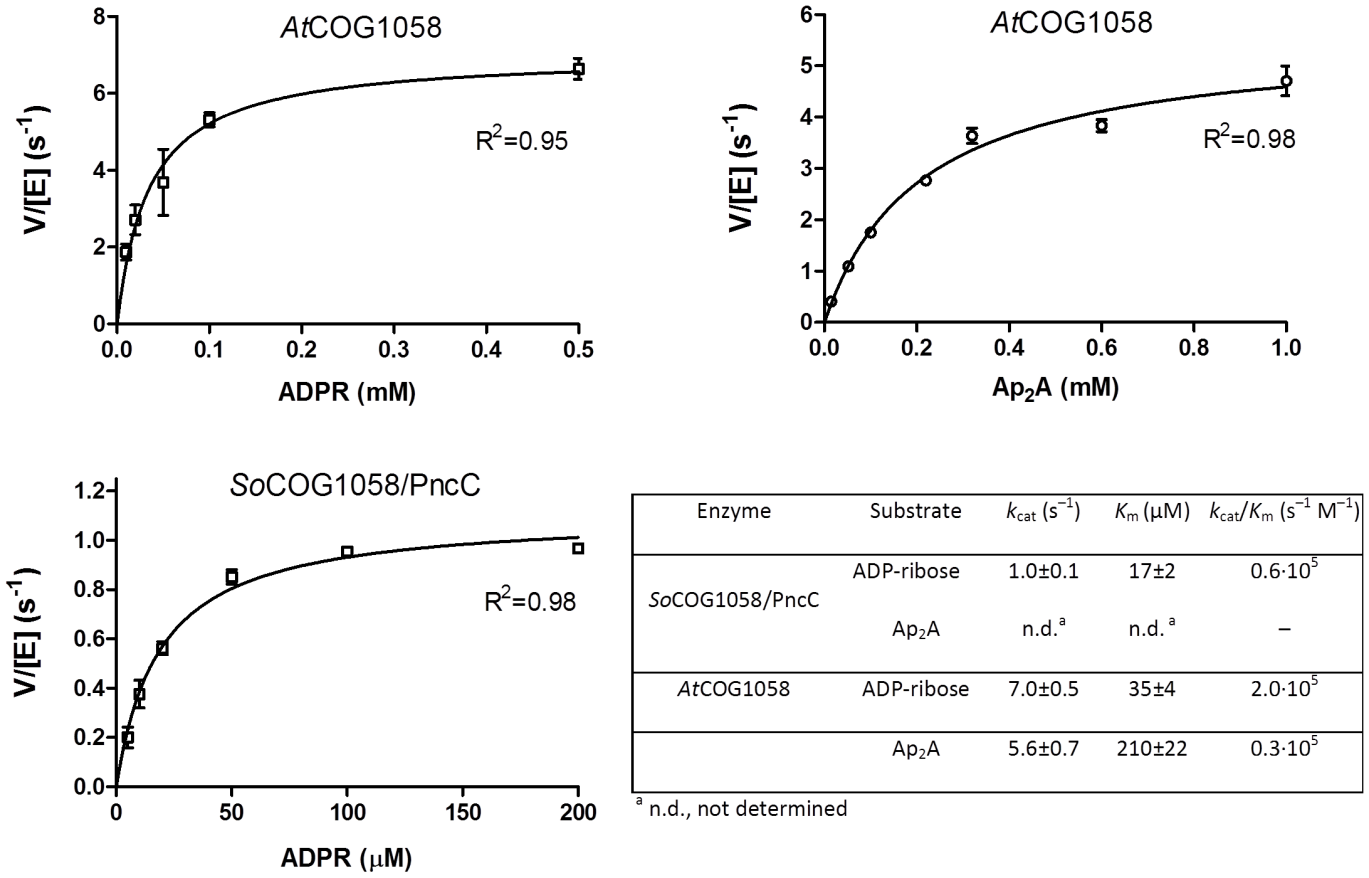
Kinetic characterization of *So*COG1058/PncC and *At*COG1058 enzymes. Plots of the initial velocities of the catalyzed reactions *versus* substrate concentrations. Kinetic parameters, calculated as described in Materials and Methods, are reported in the table.

Results of the kinetic analyses for the preferred substrates are reported in Figure 5. Although the *At*COG1058 enzyme hydrolyzes ADPR and Ap_2_A at similar rates, the catalytic efficiency (*k*_cat_/K_m_) towards ADPR is about 14 fold higher. In addition, the bifunctional enzyme is about seven-fold less efficient towards ADPR than the stand-alone pyrophosphatase.

Neither enzyme is able to remove a phosphate group from ribonucleoside mono- and diphosphates or from NADP, nor to hydrolyze the phosphodiester bond in 2′, 3′- and 3′, 5′-cyclic nucleotides (not shown).

### COG1058 Phylogenetic Analysis Reveals a Wide Distribution and Fusion with Different Catalytic Domains

**Figure 6:**
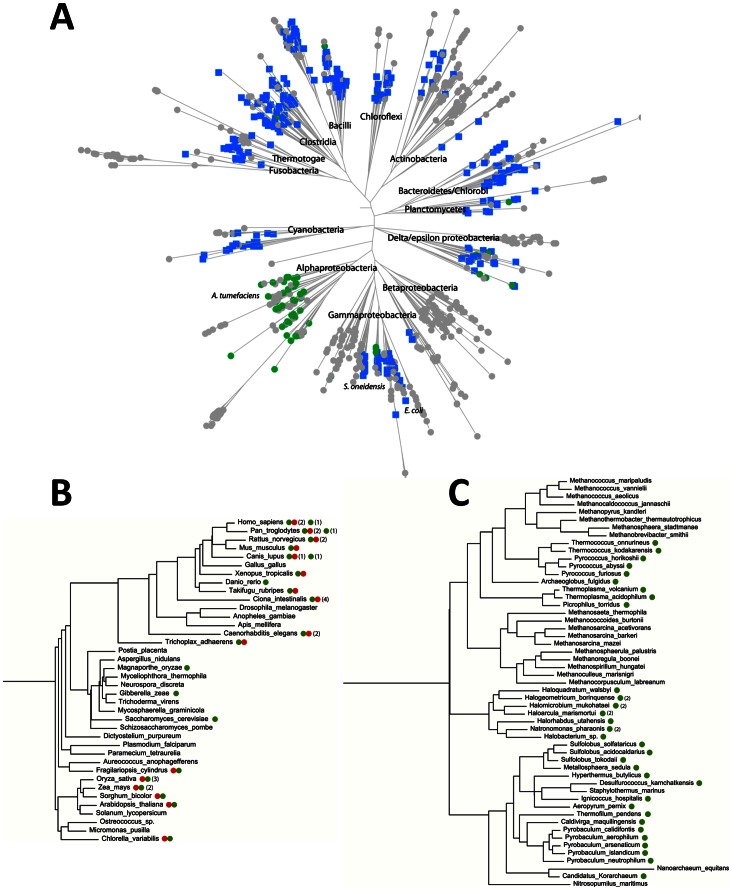
Phylogenetic distribution and domain composition of COG1058. Schematic representation of bacterial (A), eukaryotic (B) and archaeal (C) species trees showing COG1058 genes mapping. Green circle designates the COG1058 gene; the FAD synthase gene is represented by a red circle; the fused COG1058/pncC gene is shown as a blue square. Numbers within squares represents the number of gene copies per genome.

**Figure 7:**
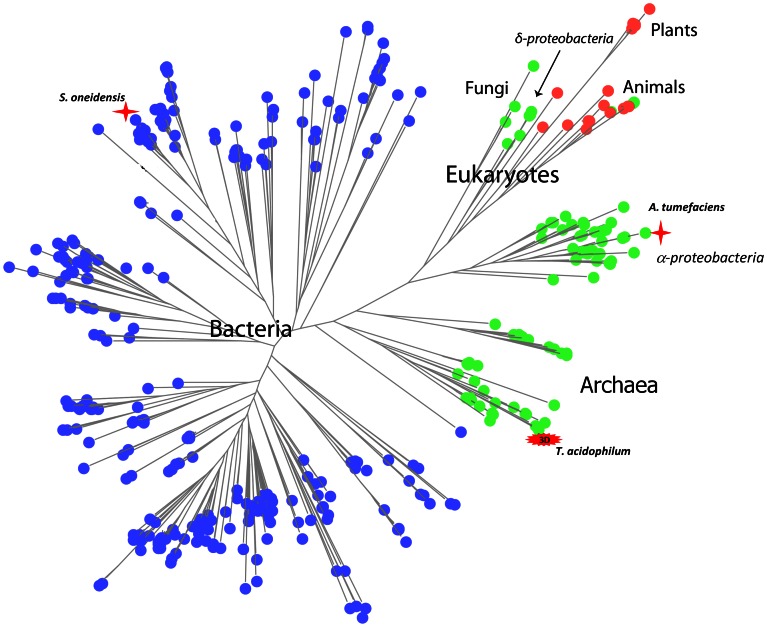
Phylogenetic tree of COG1058. Schematic representation of the COG1058 phylogenetic tree (full version is in Fig. S1). The stand-alone COG1058 gene and the gene fused with FAD synthetase and pncC genes are depicted as green, red and blue circles, respectively. The *Shewanella oneidensis* and *Agrobacterium tumefaciens* COG1058 proteins, experimentally characterized in this work, are marked by red stars. *Thermoplasma acidophilum* COG1058 protein, whose 3D structure is available, is highlighted.

Analysis of the phylogenetic distribution of COG1058 members in the three kingdoms of life revealed that they are widely distributed, occurring in approximately half of the eukaryotic, bacterial, and archaeal genomes (Figure 6). The COG1058 domain can be found either as a stand-alone domain, or in a fused form with NMN deamidase or FAD synthase (that catalyzes FMN adenylylation to FAD). In particular, in archaea, in α- and some δ-proteobacteria, the COG1058 domain occurs only as a single domain, while in remaining bacterial taxonomic groups it is mostly found fused with PncC (Figure 6A–C). Eukaryotes have both single- and two-domain proteins, the latter being composed of the COG1058 domain fused to FAD synthase (Figure 6B). While the single-domain form is mostly present in fungi, the fused form is widely distributed among plants and animals. Notably, in plants the COG1058 domain is located at the N-terminus, whereas in animals it occurs at the C-terminus. The large-scale topology of the COG1058 phylogenetic tree (Figure 7, and Figure S1) is largely consistent with the top-level topology of the species tree, i.e. the bacterial, archaeal and eukaryotic proteins form distinct clusters. This implies the absence of the horizontal transfer of the COG1058 domain between the three main kingdoms. The analysis of the tree also shows that the proteins in the α-proteobacterial group are evolutionarily closer to the eukaryotic and archaeal proteins than to their bacterial counterparts. In addition, the δ-proteobacterial group of enzymes is present in the eukaryotic branch, suggesting that it likely represents the ancestor group of the eukaryotic proteins. As to the domain composition, the two-domain bacterial proteins cluster into a homogenous group, well separated from all other forms, *i.e.* the bacterial, archaeal and eukaryotic single-domain enzymes and the eukaryotic two-domain enzymes. In this view, the COG1058 domain fused to NMN deamidase is evolutionarily distant from both the stand-alone and the FAD synthetase-fused form, the latter two forms being closely related to each other.

### Mutagenesis Guided by Multiple Sequence Alignment and Structural Analysis Reveals the Identity of Catalytic Residues

**Figure 8:**
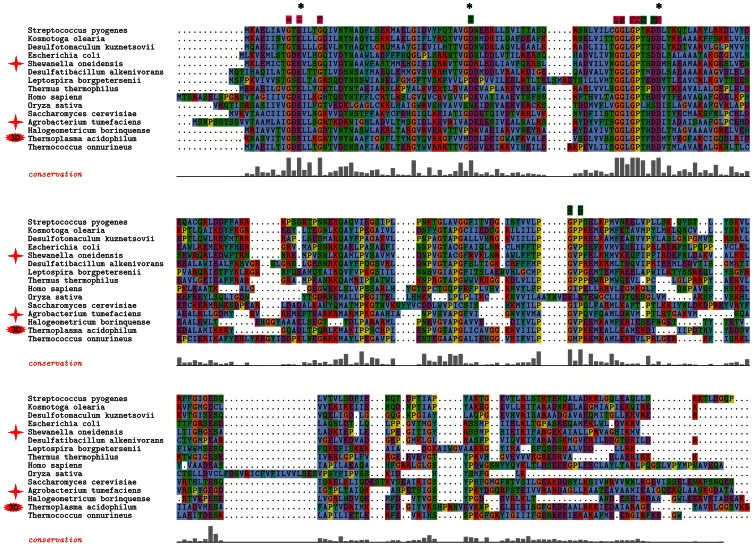
Multiple sequence alignment of selected COG1058 proteins. Multiple alignment of representative members of the COG1058 family (full version is available in Figure 2). Positions of residues conserved in all members of the family are highlighted at the top of the alignment in magenta. The residues highlighted in green are conserved in all proteins, with the exception of the plant subfamily. Residues are numbered according to the *T. acidophilum* protein. Proteins experimentally characterized in this work are marked by red stars. Residues mutated in the *A.tumefaciens* protein are marked with black asterisks.

**Figure 9:**
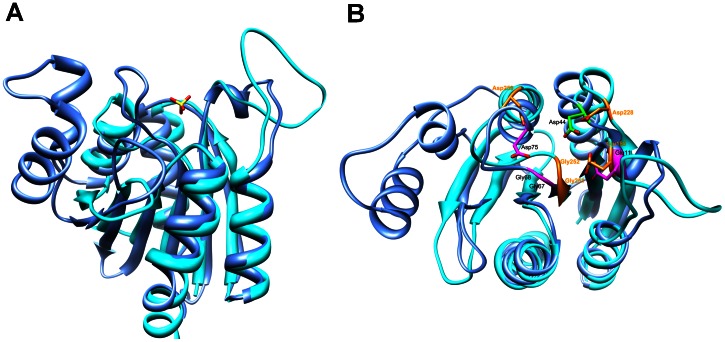
Structural comparison of *Thermoplasma acidophilum* COG1058 and *E. coli* MoeA enzymes. A) Ribbon representation of superposed *T. acidophilum* COG1058 (blue) and *E. coli* MoeA (cyan) structures. The sulfate ion found in the COG1058 structure, likely indicative of the position of the active site, is shown as ball and stick; B) Superposed COG1058 and MoeA structures viewed from the top. The MoeA acidic residues predicted to be involved in catalysis and the two glycines of the conserved motif proposed to interact with the phosphate moiety of the MPT substrate are highlighted in orange, and their superposition to identical residues in the COG1058 structure is shown.

A multiple sequence alignment using a collection of 361 bacterial, 36 archaeal, and 34 eukaryotic completely sequenced genomes, annotated in The SEED database [23], revealed that the COG1058 domain is highly conserved (Figure S2). A multiple alignment of the domain in the most divergent sequences, including the *At*COG1058 and the *So*COG1058/PncC proteins characterized in this work, is depicted in Figure 8. A total of eight residues (highlighted in magenta in Figure 8) were conserved among over 95% of the COG1058 proteins in all sequenced genomes, suggesting their likely role in protein’s function or stability. The conserved residues appear to define two putative signature motifs, namely GXEX_3_G and GGL/IGPX_3_D. In addition, five residues (highlighted in green in Figure 8) were found to be conserved in all COG1058 sequences, with the exception of the plant subfamily of proteins (Figure S2). To get an insight into the possible functions of the conserved residues, we performed a structural homology search by using as the query the available high-resolution (2 Å) crystal structure of the *Thermoplasma acidophilum* COG1058 protein (PDB ID: 3KBQ), as determined at the Midwest Center for Structural Genomics (http://www.mcsg.anl.gov/). Notably, a DALI search revealed high structural similarity (Z score >10 ) with the fold of proteins from the *E. coli* MoCo biosynthetic pathway (MogA, the domain III of MoeA, and MobA), as well as with domains of mammalian gephyrin and plant Cnx1, which are also involved in MoCo biosynthesis [30], [31], [32]. The superimposition of the 3KBQ three-dimensional structure and domain III of *E. coli* MoeA (PDB ID: 1G8L) is shown in Figure 9A. Based on both the significant overall structure homology and the similarity of the reactions catalyzed by the two proteins, we speculated that their active site might be at least partially conserved. Indeed, a high degree of structural conservation of the residues important for MoeA catalysis and highly conserved in COG1058 was evident (Figure 9B). They comprise the MoeA acidic triad Glu188, Asp228 and Asp259, which has been predicted to be involved in the catalysis by coordinating the divalent cation required for the MoeA-catalyzed reaction, as well as Gly251 and Gly252 of the SSGGVS motif, which in the MoeA-MPT model is located in proximity of the phosphate group of MPT [30], [31], [33]. As shown in Figure 9B, these residues have the same structural location as Glu11, Asp44, Asp75, Gly67 and Gly68 of the 3KBQ structure. With the exception of Asp44, which is replaced by an asparagine in the plant proteins, the superimposed 3KBQ residues are highly conserved in all COG1058 members (Figure 8 and Figure S2). The importance of the charged conserved residues was experimentally validated by performing site-directed mutagenesis on the *A. tumefaciens* enzyme. Three mutants were generated by replacing the *At* COG1058 residues Glu21, Asp54 and Asp 85 with alanine. A D54N mutant was also obtained, to determine whether such substitution, which occurs in all plant proteins, would affect the catalytic activity. All four mutants were purified and assayed for the ADPR pyrophosphatase activity (Figure S3 ). None of them resulted to be endowed with a detectable enzymatic activity, confirming their essentiality for catalysis, and suggesting that the plant COG1058 subfamily is devoid of ADPR pyrophosphatase activity.

## Discussion

In this work, we identified the bacterial members of COG1058 as novel ADPRPs, endowed with structural and catalytic properties clearly distinct from those of the ADPRPs belonging to the Nudix hydrolase family. Besides possessing a completely different fold, COG1058 ADPRPs show unique Co^+2^- and K^+^-dependence, with an optimum pH at 7.5, whereas Nudix ADPRPs are either Mg^+2^- or Mn^+2^-dependent, with a more alkaline optimum pH [34]. Nevertheless, COG1058 ADPRPs display a catalytic efficiency comparable to that of characterized bacterial Nudix ADPRPs [35], [36]. In addition, both types of ADPRPs exhibit a peculiar tendency to occur in a fused form with enzymes involved in the recycling to NAD of its by-products, suggesting a common functional connection with NAD regeneration (Figure 1). Our discovery of a novel bacterial ADPRP family reinforces the relevance of ADPR in bacteria and suggests the existence of sustained ADPR-producing processes. This is also in keeping with the finding that in bacteria the overall intracellular ADPR content, including both the free and the protein-bound forms, is in the low millimolar range [22]. It is well known that free ADPR is a highly reactive molecule that causes non-enzymatic glycation of proteins, leading to loss of function [37]; on the other hand, recent evidence in both eukaryotes and bacteria shows that ADPR acts as a cellular signal [13], [38], [39]. In particular, in bacteria, it functions as a signal of NAD pool consumption promoting transcription of NAD biosynthetic genes through its binding to the NrtR repressor [39]. In this view, ADPRPs represent not just “housecleaning enzymes”, merely scavenging potentially toxic ADPR, but also important players in ADPR signaling. In addition, their fusion to NAD-synthesizing enzymes suggests their direct contribution to NAD regeneration.

The genomic context analysis of the *ADPRP*(*COG1058)/pncC* gene shows that it is most frequently associated in predicted operons with the recombinase gene *recA* and genes coding for enzymes involved in various aspects of DNA/RNA metabolism, including RNA repair. Interestingly, in some δ-proteobacteria and in the Deinococcus/Thermus group, *ADPRP*(*COG1058)/pncC* and *recA* are associated in a polycistronic operon with the gene *ligT*, encoding the enzyme 2′-5′ RNA ligase, which is capable of joining *in vitro* yeast tRNAs splicing intermediates to form internal 2′-5′ linkages, and whose role in bacterial RNA repair has been recently postulated [40], [41]. In the Deinococcus/Thermus group, such operon is further extended to include a gene encoding a protein of the YgfZ family, known to participate in the repair of iron/sulphur clusters, hence involved in maintaining the activity of several Fe-S enzymes, including the enzyme MiaB responsible of the methylthiolation of tRNA [42]. The presence of the *ADPRP(COG1058)/pncC* gene in such operons might be indicative of the requirement of NMN deamidase and ADPRP activities during RecA-dependent processes likely involved in DNA/RNA repair. The former would scavenge NMN, a potent inhibitor of the NAD-dependent DNA ligase, ensuring, at the same time, NAD supply to the ligase reaction [10]; the ADPR activity, in turn, might be involved in the scavenging of free ADPR. Indeed, the occurrence in bacteria of ADPR-producing processes during nucleic acids repair has been recently supported by bioinformatic predictions [43]. In addition, in *Deinococcus radiodurans* cells a marked induction of the operon *ADPRP*(*COG1058)/pncC-recA-ligT* has been observed after irradiation or desiccation [41], [44], and a *Streptococcus mutans* strain deleted of *ADPRP(COG1058)/pncC* shows a significant increase in the sensitivity to DNA damaging agents [45]. Finally, the proposed role of the bacterial (COG1058)ADPRP in the scavenging of ADPR during DNA/RNA repair processes is in keeping with the marked increase of the ADP-ribosyltransferase activity observed in several bacterial species in response to DNA damage [46].

In this work we have identified as ADPRPs both the *So*COG1058 domain fused to NMN deamidase and the stand-alone *At*COG1058 domain. These results, together with those derived from the multiple sequence analysis performed on the whole COG1058 and confirmed by the mutagenesis experiments, enabled us to extend the assignment of the pyrophosphatase function to all COG1058 members, with the apparent exception of plant proteins that lack conservation of catalytic residues. COG1058 pyrophosphatase activity is in keeping with the high structural similarity of the proteins with the bacterial enzyme MoeA and its eukaryotic orthologues of the MoCo biosynthetic pathway, which catalyze a complex reaction also involving the cleavage of a pyrophosphate bond. In particular, the structural conservation of identical catalytic residues in *T. acidophilum* COG1058 and MoeA suggests a common catalytic mechanism. Given that the sequence conservation is limited to a stretch of 60 residues (30% identity), it can be hypothesized that the two families might have evolved by divergent evolution from a common ancestor [47].

The phylogenetic analysis of COG1058 showed that the eukaryotic members are evolutionarily closer to the more versatile *At*COG1058 ADPRP than to the strictly ADPR-specific *So*COG1058 enzyme, suggesting that the eukaryotic pyrophosphatases might have evolved a distinct substrate specificity. Considering that in higher eukaryotes the COG1058 domain occurs in a fused form with FAD synthase, it will be worth to investigate whether COG1058 eukaryotic pyrophosphatases might hydrolyze FAD as the preferred substrate.

## Supporting Information

Figure S1 **Phylogenetic tree of COG1058.** Color notations are the same as in Figure 7.
(PNG)

Figure S2 **Multiple alignment of COG1058 sequences.**
(TIF)

Figure S3 ***At*** **COG1058 mutants characterization.** SDS-PAGE (upper panel) of 8 µg and 0.8 µg of each purified protein. HPLC chromatograms (lower panel) of the reaction mixtures prepared as described in Materials and Methods, incubated for 10 min in the presence of 0.08 µg/ml of each protein. A control mixture, in the absence of protein, was also analyzed (thin gray line). AMP and ADPR standards were subjected to HPLC analysis in the same conditions (thin black line).
(TIFF)

Table S1 **Sequences of oligonucleotides used as primers for cloning and mutagenesis.**
(DOCX)
