# Supplementary figures and images for: Genomics-Guided Analysis of NAD Recycling Yields Functional Elucidation of COG1058 as a New Family of Pyrophosphatases
Source: PLoS One. 2013 Jun 12;8(6):e65595. doi: 10.1371/journal.pone.0065595 (PMC3680494; doi:10.1371/journal.pone.0065595)

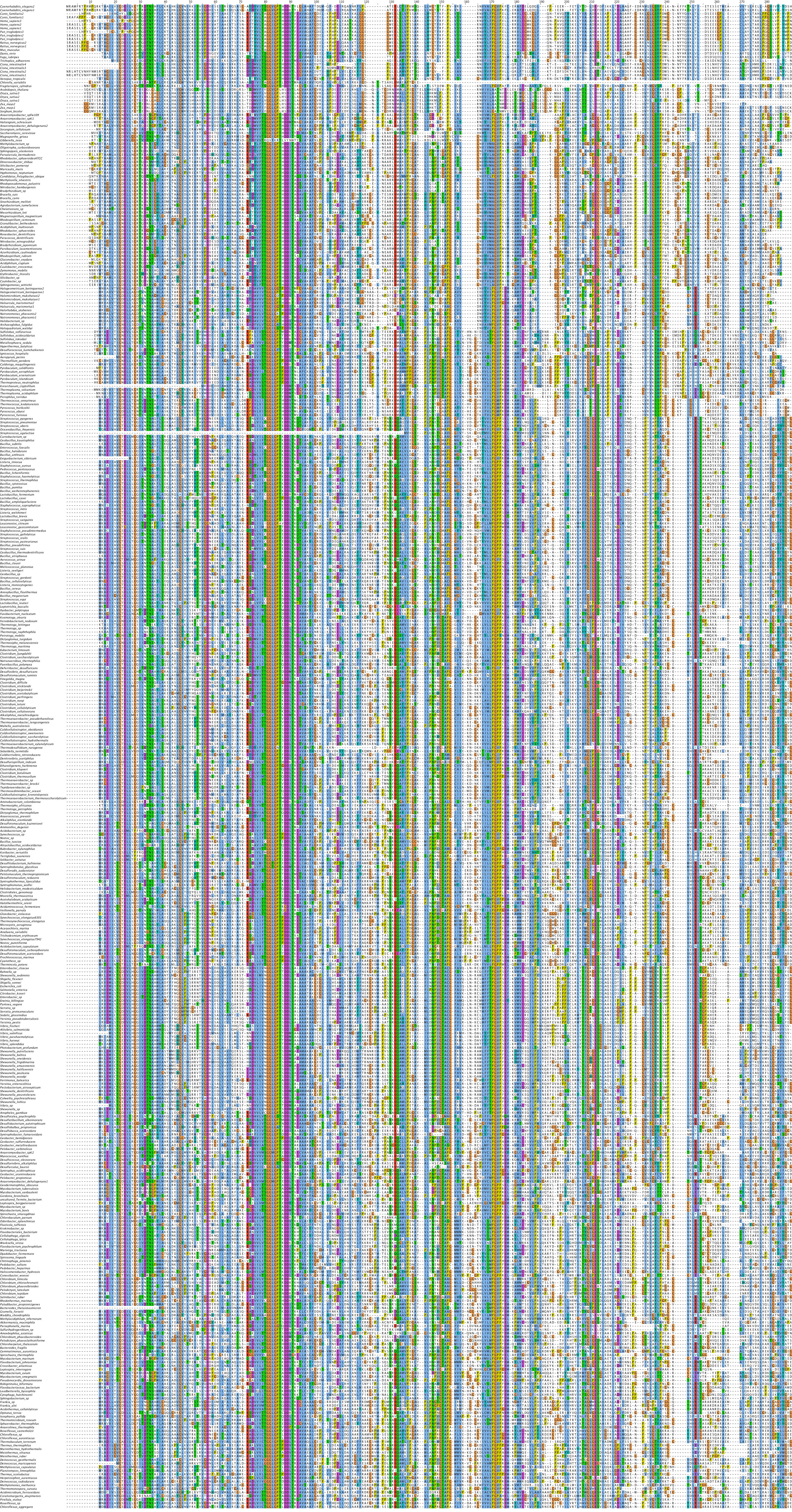

Supplement: Figure S1 — Phylogenetic tree of COG1058. Color notations are the same as in Figure 7. (PNG) [file pone.0065595.s001.png]

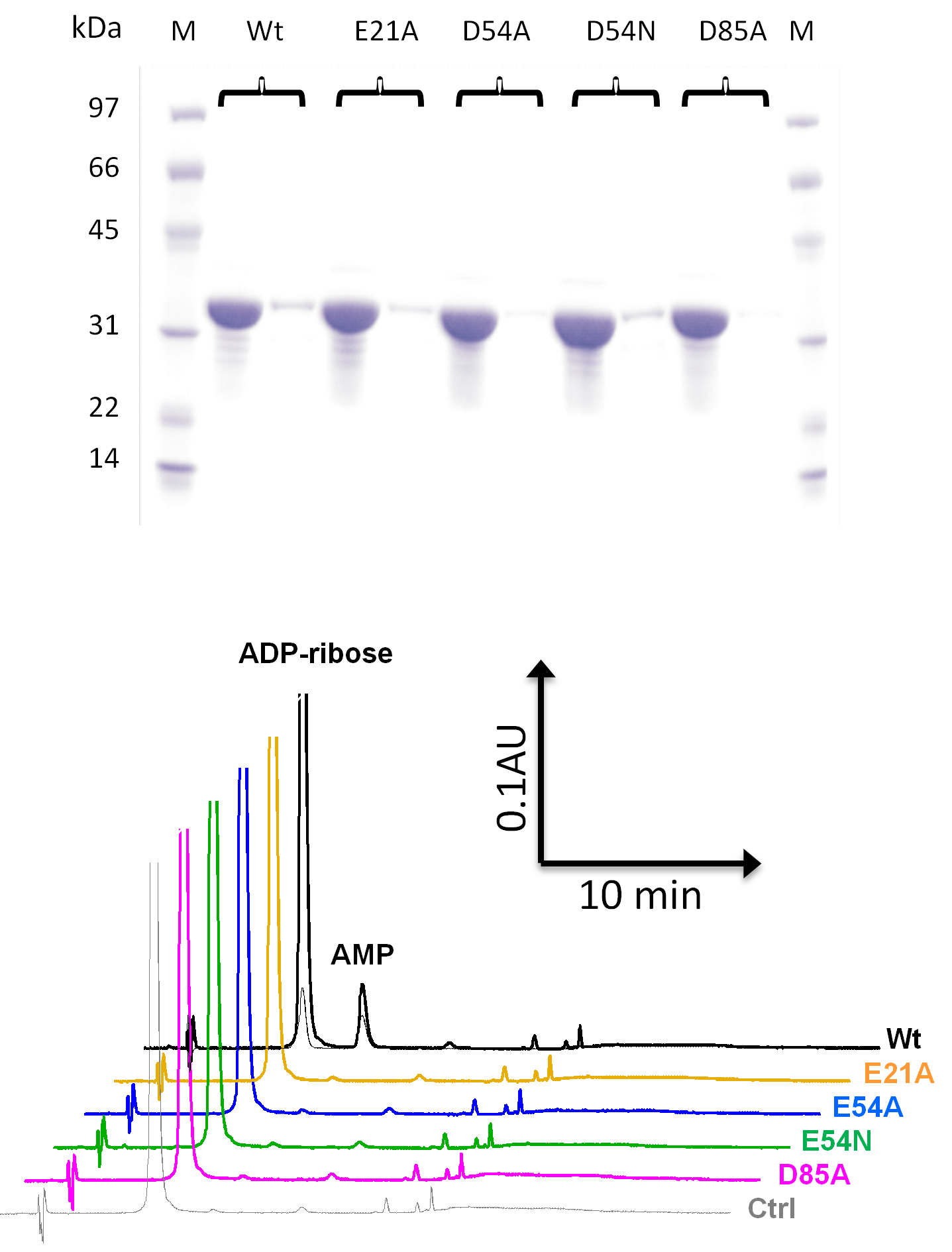

Supplement: Figure S2 — Multiple alignment of COG1058 sequences. (TIF) [file pone.0065595.s002.tif]

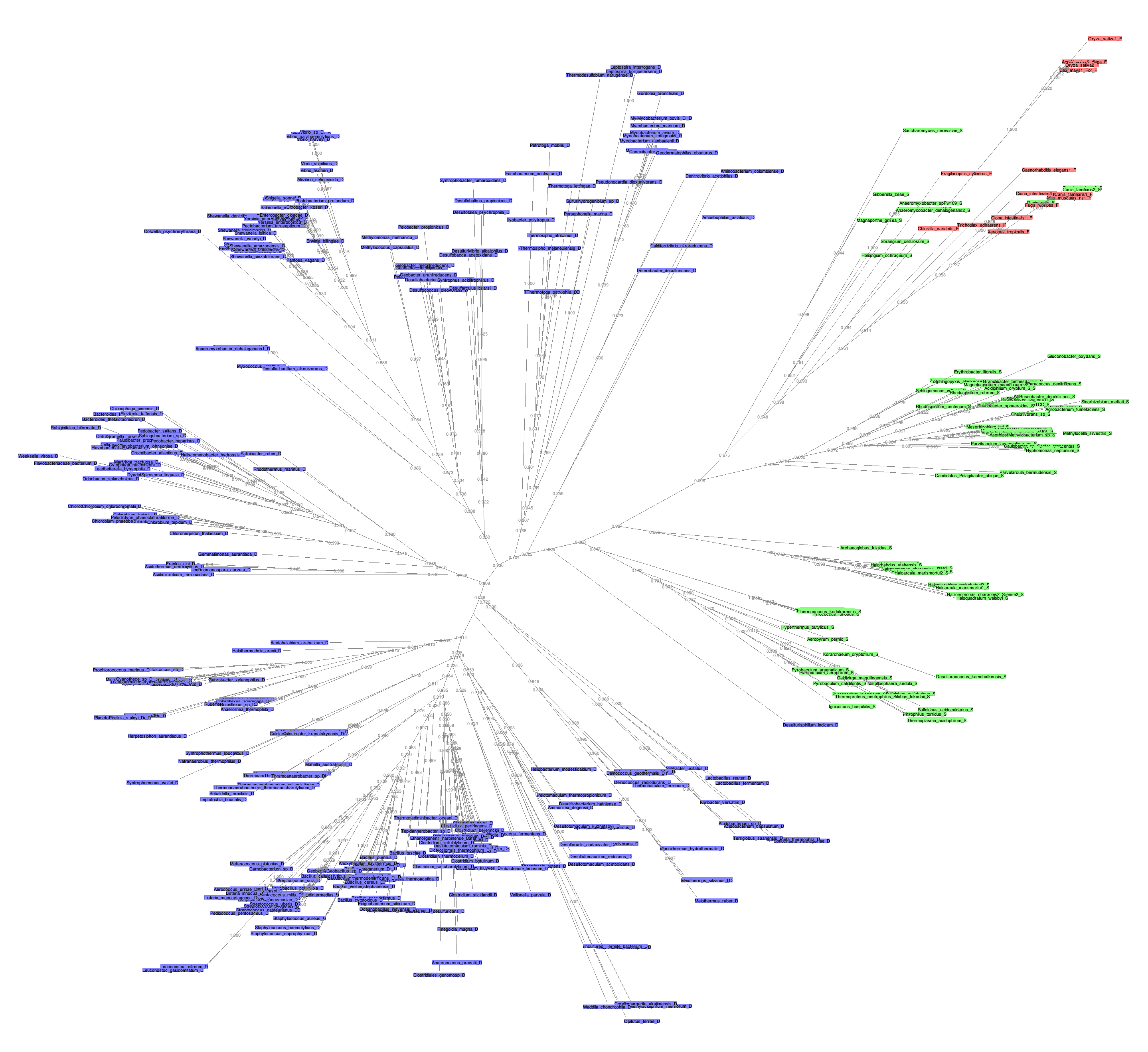

Supplement: Figure S3 — At COG1058 mutants characterization. SDS-PAGE (upper panel) of 8 µg and 0.8 µg of each purified protein. HPLC chromatograms (lower panel) of the reaction mixtures prepared as described in Materials and Methods, incubated for 10 min in the presence of 0.08 µg/ml of each protein. A control mixture, in the absence of protein, was also analyzed (thin gray line). AMP and ADPR standards were subjected to HPLC analysis in the same conditions (thin black line). (TIFF) [file pone.0065595.s003.tif]
