# Supplementary material for: Genomics-Guided Analysis of NAD Recycling Yields Functional Elucidation of COG1058 as a New Family of Pyrophosphatases
Source: PLoS One. 2013 Jun 12;8(6):e65595. doi: 10.1371/journal.pone.0065595 (PMC3680494; doi:10.1371/journal.pone.0065595)
Supplement: Table S1 — Sequences of oligonucleotides used as primers for cloning and mutagenesis. (DOCX) [file pone.0065595.s004.docx]

**Table 1. Primers used for gene cloning and mutant construction**

| Primer | Sequence | Utilization |
| --- | --- | --- |
| *Atu*COG1058 fw | CACCATGTCCAATCCCTCCTCCACCTCC | Cloning of *A. tumefaciens* COG1058 in pET100/D-TOPO |
| *Atu*COG1058 rev | TCAGGCGGTTGCATCGCCCTTGCTTGC | Cloning of *A. tumefaciens* COG1058 in pET100/D-TOPO |
| E21A fw | CCATCGGCGACGCACTGCTGTCCGG | Mutant generation |
| E21A rev | CCGGACAGCAGTGCGTCGCCGATGG | Mutant generation |
| D54A fw | CGCATCGTCGCCGCCGAGGAGGAATCG | Mutant generation |
| D54A rev | CGATTCCTCCTCGGCGGCGACGATGCG | Mutant generation |
| D54N fw | CCGCATCGTCGCCAACGAGGAGGAATC | Mutant generation |
| D54N rev | GATTCCTCCTCGTTGGCGACGATGCGG | Mutant generation |
| D85A fw | CCGACCCATGACGCCATCACCGCGGAC | Mutant generation |
| D85A rev | GTCCGCGGTGATGGCGTCATGGGTCGG | Mutant generation |
